# Comparative Transcriptomics Analyses in Livers of Mice, Humans, and Humanized Mice Define Human-Specific Gene Networks

**DOI:** 10.3390/cells9122566

**Published:** 2020-11-30

**Authors:** Chengfei Jiang, Ping Li, Xiangbo Ruan, Yonghe Ma, Kenji Kawai, Hiroshi Suemizu, Haiming Cao

**Affiliations:** 1 Cardiovascular Branch, National Heart, Lung and Blood Institute, National Institutes of Health, Bethesda, MD 20892, USA; cheng-fei.jiang@nih.gov (C.J.); ping.li@nih.gov (P.L.); xiangbo.ruan@nih.gov (X.R.); yonghe.ma@nih.gov (Y.M.); 2 Pathology Analysis Center, Central Institute for Experimental Animals, 3-25-12 Tonomachi, Kawasaki-ku, Kawasaki 210-0821, Japan; kawai@ciea.or.jp; 3 Laboratory Animal Research Department, Biomedical Research Laboratory, Central Institute for Experimental Animals, 3-25-12 Tonomachi, Kawasaki-ku, Kawasaki 210-0821, Japan; suemizu@ciea.or.jp

**Keywords:** nonalcoholic fatty liver disease (NAFLD), nonalcoholic steatohepatitis (NASH), humanized mice, liver

## Abstract

Mouse is the most widely used animal model in biomedical research, but it remains unknown what causes the large number of differentially regulated genes between human and mouse livers identified in recent years. In this report, we aim to determine whether these divergent gene regulations are primarily caused by environmental factors or some of them are the result of cell-autonomous differences in gene regulation in human and mouse liver cells. The latter scenario would suggest that many human genes are subject to human-specific regulation and can only be adequately studied in a human or humanized system. To understand the similarity and divergence of gene regulation between human and mouse livers, we performed stepwise comparative analyses in human, mouse, and humanized livers with increased stringency to gradually remove the impact of factors external to liver cells, and used bioinformatics approaches to retrieve gene networks to ascertain the regulated biological processes. We first compared liver gene regulation by fatty liver disease in human and mouse under the condition where the impact of genetic and gender biases was minimized, and identified over 50% of all commonly regulated genes, that exhibit opposite regulation by fatty liver disease in human and mouse. We subsequently performed more stringent comparisons when a single specific transcriptional or post-transcriptional event was modulated in vitro or vivo or in liver-specific humanized mice in which human and mouse hepatocytes colocalize and share a common circulation. Intriguingly and strikingly, the pattern of a high percentage of oppositely regulated genes persists under well-matched conditions, even in the liver of the humanized mouse model, which represents the most closely matched in vivo condition for human and mouse liver cells that is experimentally achievable. Gene network analyses further corroborated the results of oppositely regulated genes and revealed substantial differences in regulated biological processes in human and mouse cells. We also identified a list of regulated lncRNAs that exhibit very limited conservation and could contribute to these differential gene regulations. Our data support that cell-autonomous differences in gene regulation might contribute substantially to the divergent gene regulation between human and mouse livers and there are a significant number of biological processes that are subject to human-specific regulation and need to be carefully considered in the process of mouse to human translation.

## 1. Introduction

The liver occupies a critical role in metabolic homeostasis, and metabolic diseases of the liver, in particular nonalcoholic fatty liver disease (NAFLD) and nonalcoholic steatohepatitis (NASH), have become major global causes of liver-related morbidity and mortality [1]. The pathophysiology of the human liver, however, remains incompletely understood hindering the development of effective therapies for these debilitating diseases. Due to the intrusive nature of liver biopsy, the study of human liver physiology heavily relies on the usage of animal models particularly mouse. Although mouse-based studies have led to the development of novel therapeutics for human diseases, their translation rate remains very low [2,3], suggesting there are major differences in physiological responses between the two species. More critically, many of these differences have not been identified or carefully characterized and the lack of this critical knowledge sometimes has severe consequences. For example, a clinical trial of anti-hepatitis B drug candidate, fialuridine (FIAU), led to five deaths due to strong human-specific hepatoxicity which cannot be detected in mice, dogs, and even monkeys [4,5,6]. Clearly, we need to better understand the similarity and divergence of the pathophysiology between human and mouse livers so we can better utilize mice to model human diseases and at the same time identify critical areas where mouse is not a good model for human physiology.

One approach to systematically probe the physiological differences between human and mouse livers is to study their global gene expression. Indeed, with the rapid development of next-generation RNA sequencing technologies, we can now compare gene expression at an unprecedented throughput and depth, and recent comparative analyses have identified a substantial number of differentially regulated genes between human and mouse livers [7,8]. However, an important unresolved conceptual question regarding this differential gene regulation is whether they are largely caused by the differences in environment factors such as diet, exercise, etc., or a sizable fraction of them are the results of cell-autonomous difference in gene regulation in human and mouse liver cells. It is fundamentally important to differentiate the two possibilities as the former represents areas where current mouse models can be improved to better model human pathophysiology whereas the latter constitutes human-specific regulation that can only be adequately studied in a human or humanized system.

In this work, we performed a series of comparative analyses of gene regulation in human and mouse livers under increasingly stringent conditions to eliminate the influence of factors external to liver cells and used informatic approaches to define gene networks that are physiologically relevant. We found that there are a surprisingly large number of genes that exhibit completely opposite regulation by fatty liver disease in human and mouse, a pattern that persists in additional closely matched in vitro and in vivo conditions. Most critically, a high percentage of oppositely regulated human and mouse genes also exist in a humanized liver where human and mouse cells share an identical in vivo environment. Taken together, our work support that a substantial fraction of differentially regulated genes between human and mouse are very likely the result of cell-autonomous mechanisms and they need to be carefully considered in mouse to human translation.

## 2. Materials and Methods

### 2.1. RNA-Seq Analysis for Public Data

The human male nonalcoholic fatty liver disease (NAFLD; normal weight *n* = 14, NAFL *n* = 12) and HFD fed mouse liver RNA-seq datasets (standard chow *n* = 98, high fat diet *n* = 94) were downloaded from Sequence Read Archive (SRA) database (https://www.ncbi.nlm.nih.gov/geo/, accession number: SRP186450 and SRP063455 and the sample information details can be found in Supplementary Table S4). SRA files were unzipped by fastq-dump (sratoolkit/2.9.6) and were trimmed by cutadapt/2.8. After quality control by FastQC/0.11.8, the trimmed fastq files were aligned by HISAT2 to human GENCODE v32 or mouse GENCODE M23 genome reference, respectively. The aligned reads were calculated by featureCounts (subread/2.0, http://subread.sourceforge.net/) and differential expression genes (DEGs) were analyzed by R package DESeq2/3.1.0 (https://bioconductor.org/packages/release/bioc/html/DESeq2.html). The statistical information for differential expression analysis can be found in Supplementary Table S5.

### 2.2. Microarray Analysis for Public GEO Data

The microarray data for human and mouse primary hepatocytes treated with PPARα agonist Wy14643 (GSE17251 and the sample information details can be found in Supplementary Table S4) [9] were downloaded by R package GEOquery/3.6 (http://bioconductor.org/packages/release/bioc/html/GEOquery.html). For each gene, we kept the probe expression values which had the maximum mean value over all samples. The DEGs were analyzed by R package limma/3.10 (http://bioconductor.org/packages/release/bioc/html/limma.html) using the paired analysis method.

### 2.3. Multiscale Embedded Gene Co-Expression Network Analysis (MEGENA)

The human NAFLD and mouse HFD RNA-seq data were normalized by DESeq2 VST methods and the ODEGs expression matrix were sent to MEGENA analysis to generate the enriched functional modules using R packages MEGENA/1.3.7 (https://cran.r-project.org/web/packages/MEGENA/index.html) [10]. Modules were intersected with the most significant DEGs and the other module gene sets using gene symbol ids and the significance level of which was determined by Fisher’s exact test [11].

### 2.4. Adenovirus Production

The adenovirus was produced using pAD/Block-it system (Invitrogen, Carlsbad, CA, USA) according to the manufacturer’s protocols and were purified byCsCl gradient centrifugation. The purified adenovirus was desalted with PD10 columns (GE Healthcare Life Sciences, Marlborough, MA, USA) and titered with Adeno-X Rapid Titer Kit (Clontech, Mountain View, CA, USA) which was previously described [12]. The shRNAs were designed using the following sequences: human HuR shRNA: GGCTTTGTGAC CATGACAA; mouse HuR shRNA: GGTTTGGGCGAATCATCAA).

### 2.5. Animal Experiments

All animal experiments were performed in accordance and with approval from the NHLBI Animal Care (ethic approval number: H-0248) and Use Committee or the Animal Care Committee of the Central Institute for Experimental Animals (CIEA, Kanagawa, Japan). The humanized mice and wild type (WT) mice were prepared as previously described [12,13]. In brief, for fasting–refeeding experiment the humanized TK-NOG mice were allowed free access to food (Fed; *n* = 4) or subjected to a 24 h food withdrawal (Fasting; *n* = 5) or subjected to a 24 h food withdrawal followed by a 4 h refeeding (Refeeding; *n* = 5) before tissue harvest. For WT mice, male C57BL/6 (B6) mice were purchased from Jackson Laboratory (Bar Harbor, ME, USA) at 8 weeks of age and were acclimatized to the housing for 10–14 days before experiments.

HuR shRNA adenoviruses were delivered into WT or humanized mice intravenously at 2 × 10^9^ pfu/mouse for both control virus (humanized mice *n* = 4; WT mice *n* = 6) and HuR shRNA virus (humanized mice *n* = 4; WT mice *n* = 6). After seven days, liver tissue samples were harvested after a 4 h fasting and stored immediately in liquid nitrogen till further analysis.

### 2.6. RNA Extraction and RNA-Seq Analysis

The frozen liver tissue samples were homogenized in Trizol reagent (Invitrogen) using TissueLyser LT system (Qiagen, Valencia, CA, USA). The isolated RNA was purified by MagMAX RNA Extraction Kit (Thermo Fisher Scientific, Waltham, MA, USA) and the construction of strand specific sequencing libraries using TruSeq Stranded Total RNA Prep kit (Illumina, San Diego, CA, USA) and the sequencing was performed at NHLBI DNA Sequencing and Genomics Core using Illumina HiSeq 3000 paired-end sequencing platform. The raw fastq files were trimmed by cutadapt/2.8. After quality control by FastQC/0.11.8, the trimmed fastq files were aligned by HISAT2 to mouse GENCODE M23 genome reference for WT mice. For the alignment of RNA-seq data of the chimeric livers from humanized mice, we established the genome reference index by combining both human and mouse genome sequence and renamed the annotation by adding the prefix as “human_” or “mouse_” dependent on the source organism using the annotation versions by human GENCODE v32 and mouse GENCODE M23 [12]. The aligned reads were calculated by featureCounts (subread/2.0) and differential expression genes (DEGs) were analyzed by R package DESeq2/3.1.0. The statistical information for differential expression analysis can be found in Supplementary Table S5.

### 2.7. Human and Mouse Divergence Analysis

The expression correlation and divergence were analyzed between human and mouse homology genes (MGI, http://www.informatics.jax.org/homology.shtml). The divergence signatures for DEGs between human and mouse were defined as consistent, logFC(mouse) × logFC(human) > 0; opposite, logFC(mouse) × logFC(human) < 0; inconsistent, defined as DEGs (*p* < 0.05) either in human or mouse, but not both. The expression correlation plots used the logFC(human) and logFC(mouse) as input data and their correlation was analyzed by the Pearson’s method. The divergence score for consistent or opposite genes between human and mouse were defined as |logFC(human)−logFC(mouse)|, and the plots were drawn by R package ggpubr/0.2.4 (https://cran.r-project.org/web/packages/ggpubr/index.html).

### 2.8. Pathway Enrichment Analysis

#### 2.8.1. Gene Ontology (GO) Enrichment Analysis

The DEGs (normal *p* < 0.05) were sent to GO Biological Process (BP) using R package clusterProfiler/3.14.3 (https://bioconductor.org/packages/release/bioc/html/clusterProfiler.html) using default settings [14]. The top enriched pathways were visualized by function dotplot and cnetplot, respectively.

#### 2.8.2. Gene Set Enrichment Analysis (GSEA)

The list of more significant DEGs (|logFC > 0.4|, padj < 0.05) in human and mouse were ranked by fold change, respectively, and the GO BP pathway enrichments were analyzed by GSEA_4.0.3. The top 200 enriched pathways for human DEGs were selected and the normalized enriched scores (NES) were plotted together with the ones for the same enriched pathways for mouse DEGs.

#### 2.8.3. Gene Set Variation Analysis (GSVA)

The RNA-seq data for human and mouse were normalized by DESeq2 to get DEGs. The consistent and opposite DEGs between human and mouse were isolated, their normalized gene expression data for each sample were analyzed using R package GSVA/1.34.0 (https://www.bioconductor.org/packages/release/bioc/html/GSVA.html) [15] to calculate the enrich score for the pathways, which were then visualized by heatmap using R package/1.0.12.

### 2.9. Data Availability

RNA-seq dataset “HuR knockdown in humanized and wildtype mice liver samples” (GSE161462) and “Fasting Fed RNA sequencing experiment of mice with humanized livers” (GSE126587) can be assessed at GEO Database.

## 3. Results

### 3.1. Differential Gene Regulation in Models of Fatty Liver Disease between Human and Mouse

**Figure 1:**
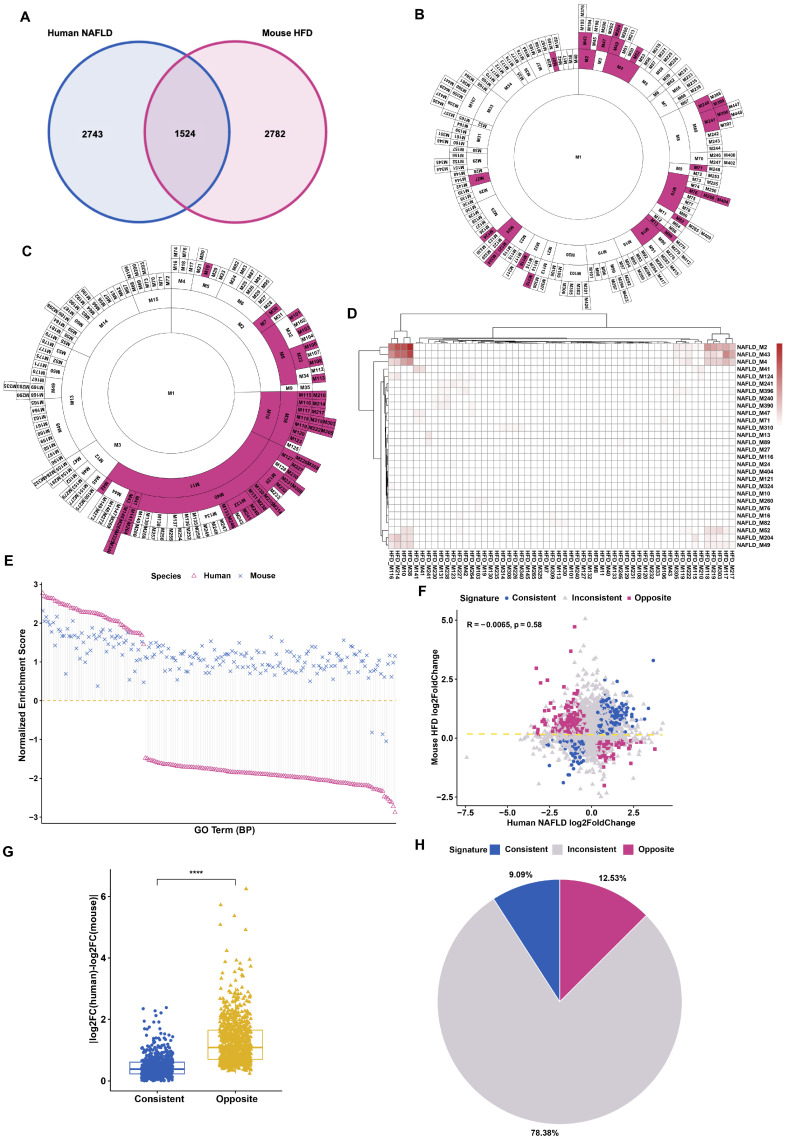
Distinct gene regulation in model disease between human and mouse. (**A**) Venn diagram of the overlapped differentially expressed genes (adjusted *p* value < 0.05) in fatty liver disease between human nonalcoholic fatty liver disease (NAFLD) and mouse high fat diet (HFD). (**B**,**C**) The coexpressed modules for human and mouse genes were generated by Multiscale Embedded Gene co-Expression Network Analysis (MEGENA). Fisher’s exact test was employed to evaluate the overlap significance between more significant differentially expressed genes (DEGs; padj < 0.05, |logFC| > 0.4) and the module genes using gene symbols and the representative modules enriched with DEGs (FET *p*-value < 0.05) were labeled as red. (**D**) The similarity between human and mouse representative modules. The significance level of the overlap between two modules was determined by Fisher’s exact test. (**E**) GSEA GO term analysis for more significant DEGs (*p* adjust value < 0.05, |logFC| > 0.4) in human and mouse fatty liver disease, showing the shared terms. (**F**) Correlation analysis of the log2 (fold change) for the DEGs (*p* adjust value < 0.05) in human and mouse fatty liver disease. Consistent genes mean log2 (fold change) human × log2 (fold change) mouse > 0; opposite genes mean log2 (fold change) human × log2 (fold change) mouse < 0; inconsistent genes are only differentially expressed either in human or mouse fatty liver disease. (**G**) The divergence (|log2 (human fold change) − log2 (mouse fold change)|) of log2 (fold change) values for consistent and opposite genes between human and mouse. Data represent mean ± SEM, **** *p* < 0.0001, two-tailed unpaired Student’s *t*-test. (**H**) The percentages of consistent, opposite, and inconsistent genes.

This work aims to test a concept, i.e., if there are substantial cell-autonomous differences in gene regulation in human and mouse liver cells under a physiologically relevant setting. As improving the quality of human life is the ultimate goal of biomedical research, we used the human population as the default reference in this analysis and tried our best to identify mouse cohorts that could match the human conditions as closely as possible. Previous comparative analyses have revealed a significant number of differentially regulated genes in human and mouse livers [7,8] but these comparisons were often made between humans of diverse genetic backgrounds and inbred mice of a pure strain. In addition, male mice have been traditionally used for metabolic studies whereas human samples were often a mixture of both males and females who can sometimes exhibit striking differences in their metabolic responses [16]. To minimize the impact of the inequivalence of gender and genetic complexity during comparison, we compare the regulation of liver gene expressions by fatty liver disease in human and mouse samples which are composed of diverse genetic makeups and the same gender. Specifically, we analyzed the regulation of hepatic gene expressions in humans with nonalcoholic fatty liver disease (NAFLD) [17] and outbred mice with high fat diet (HFD)-induced fatty liver [18] as compared to their normal controls for all males. All homologous genes passing adjusted *p* values of 0.05 were initially considered as observational differentially expressed genes (DEGs). There were totally 4267 and 4306 regulated genes in human and mouse, respectively. The fatty liver associated gene expression in human and mouse were very different, only 1524 genes were commonly regulated with 5525 genes showing nonoverlapping or inconsistent regulation, i.e., regulated only in either human or mouse (Figure 1A).

As gene networks rather than individual genes govern physiological responses, we employed MEGENA (Multiscale Embedded Gene co-Expression Network Analysis) to characterize functionally coexpressed gene modules associated with fatty liver disease using the observational DEGs as the inputs. MEGENA identified 202 modules for human NAFLD and 186 for mouse HFD (Supplementary Tables S1 and S2). Next, we identified the modules with the enrichment of the more significant DEGs (FDR < 0.05, |logFC| > 0.4) (Figure 1B,C), which represent the signature genes of human NAFLD and mouse HFD, respectively. To examine the similarity of these signature genes enriched modules between human and mouse, the overlapping patterns between human and mouse modules were visualized (Figure 1D), showing very poor overlaps. These data highlight the vast differences in the liver gene expression network between human NAFLD disease and mouse HFD model.

To further ascertain the differences in biological function changes associated with the DEGs between human and mouse fatty liver diseases, we performed gene set enrichment analysis (GSEA) of biological process GO terms using the fold change information of the most significant genes, to define the regulated biological functions by DEGs. We used the top 200 enriched pathways generated from human NAFLD DEGs and matched them to the enriched pathways from mouse HFD DEGs. The normalized enrichment score (NES) represents the direction of expression changes of the enriched DEGs (NES > 0, positive regulation; NES < 0, negative regulation; (NES (human) × (NES) mouse < 0 means opposite regulation). Interestingly, GSEA results indicated that human and mouse livers exhibit a significant number of oppositely regulated signal pathways (Figure 1E and Supplementary Table S3). For example, the NES of GO_REGULATION_OF_ALCOHOL_BIOSYNTHETIC_PROCESS pathway is 1.98 (*p* value = 0.020) for human while for mouse is −1.91 (*p* value = 0.038), suggesting this pathway is oppositely regulated by fatty liver disease in human and mouse.

Given that pathway analyses support that very different biological processes were regulated by fatty liver disease in human and mouse, we took a closer look into how the observational genes were regulated by both models. Out of 7049 orthologue genes that were regulated either in human or mouse, there were 883 genes that displayed opposite fold change direction (logFC(human)× logFC(mouse) < 0) between human and mouse whereas 641 consistent genes with the same fold change direction (Figure 1F). We also calculated the absolute fold change deviation (|logFC(human) − logFC(mouse)|) which clearly illustrates the significant higher divergence between human and mouse of the opposite gene group than the consistent group (Figure 1G). Thus, the distinct gene expression in fatty liver disease between human and mouse is not only reflected in the inconsistent genes (78.38%), but also in the oppositely regulated genes (12.53%) in human and mouse (Figure 1H). When another mouse fatty liver disease model fed with CDAHFD (choline-deficient, L-amino acid-defined, high-fat diet) was used for the comparison with human NAFLD [19], albeit not outbred mice, striking patterns of opposite regulation were consistently observed (Figure S1A–D).

Thus, even after the impact of genetic and sex biases was minimized, there are still a strikingly high number of oppositely regulated genes between human and mouse livers, suggesting that some of them might be rooted in intrinsic differences in gene regulation between human and mouse.

### 3.2. Distinct Response in Gene Expression between Human and Mouse under Comparable Conditions

Fatty liver is a complex metabolic disorder that entails a multitude of pathological triggers and its initiation and progression in NAFLD patients and HFD-fed mice could be very different. Therefore, it is not a complete surprise that there are a very large number of differentially regulated genes between the two models. To further investigate the concept if some of the differential gene expressions are caused by cell-autonomous differences in gene regulation between human and mouse liver cells, we compared hepatic gene regulation in response to a single well-defined stimulus under comparable experimental settings for human and mouse systems, and to better understand the molecular underpinnings of any differential regulation, we compared gene regulation at transcriptional and post-transcriptional levels.

We first analyzed a dataset to compare gene expressions in primary human and mouse hepatocytes which were cultured under a similar condition and both treated with an agonist of PPARα (Wy14643) [9], an important liver transcriptional factor in fasting response. Corroborating our findings in fatty liver disease models, very different gene regulation by PPARα activation in human and mouse hepatocytes was observed, with more than 1800 inconsistently regulated genes, and only 204 commonly regulated genes. For the latter, in addition to 153 consistently regulated genes, 51 genes (25%) were regulated by PPARα in opposite direction in human and mouse hepatocytes, displaying higher divergence of expression changes between human and mouse than the consistent gene group (Figures S1B and S2A).

Next, we tried to define the biological functions of the consistent and opposite genes by GO term analysis. The GO term BP analysis results showed that, for both human and mouse cells treated with PPARα agonist, consistent genes were enriched in the lipid metabolism related pathway with the same directions (Figure S2C). However, oppositely regulated genes in human and mouse displayed much different enrichment patterns and even opposite enrichment. Remarkably, more than one quarter of the top 20 opposite enriched pathways presented the opposite enrichment patterns between human and mouse with more than half inconsistently enriched pathways (Figure S2D). These data indicated that there are also a significant number of oppositely regulated genes in human and mouse hepatocytes cultured under comparable conditions in response to a single acute treatment that specially activates transcriptional factor PPARα.

**Figure 2:**
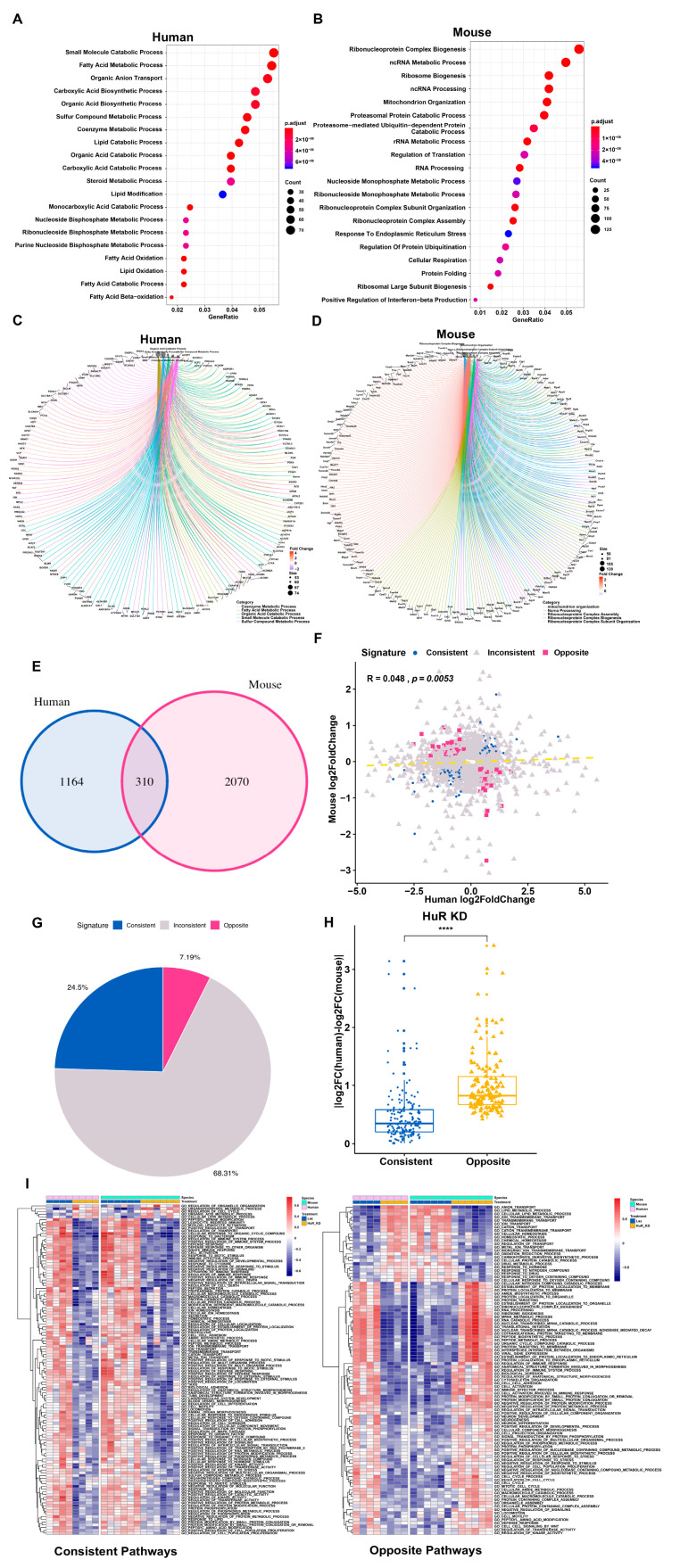
Distinct response in gene expression between human and mouse under comparable conditions. (**A**,**B**) Top 20 enriched GO term pathways for DEGs in conventional or humanized mouse liver with knockdown (KD) of mouse or human HuR, respectively. (**C**,**D**) Central network plot for the most significantly enriched pathways and their genes associated with HuR KD in conventional or humanized mouse liver. (**E**) Venn diagram of the overlapped DEGs (*p* < 0.05) between human and mouse associated with hepatic HuR KD. (**F**) Correlation analysis of the log2 (fold change) for the DEGs associated with HuR KD in conventional or humanized mouse liver. (**G**) The percentages of consistent, opposite, and inconsistent genes. (**H**) Divergence box plot for human and mouse consistent and opposite genes in HuR KD. (**I**) GSVA enrichment score heatmap of consistent and opposite genes in human and mouse HuR KD, respectively.

We next asked if differential gene regulation in human and mouse liver cells also occurs at post-transcriptional level, and to assess the physiological significance of these regulation, we compared gene regulation in human and mouse liver cells under a similar in vivo condition. We employed a mouse model with humanized liver where the mouse liver was repopulated with transplanted hepatocytes to carry out this study [13]. We knocked down HuR, a post-transcriptional regulator of gene expression, by administrating adenovirus carrying shRNAs for mouse or human HuR into conventional mice and humanized mice, respectively (Figure S3A,B). Interestingly, for the top pathways enriched for DEGs upon HuR knockdown, the human ones identified in humanized mouse liver displayed a striking association with lipid metabolism whereas the mouse ones in conventional mouse liver showed remarkable connection to RNA metabolism (Figure 2A–D). Indeed, among the total 3544 differentially expressed human and mouse genes with HuR knockdown in humanized and conventional mouse livers, only 310 were commonly regulated, whereas 3234 were inconsistently regulated between human and mouse (Figure 2E). Even in those commonly regulated genes, there were only 171 consistently regulated genes with 139 genes oppositely regulated, indicating that more than 75% of the differentially expressed genes exhibited different regulation patterns and huge divergence in the two species (Figure 2G,H). To further analyze biological functions of the consistent and opposite genes, we performed GO term GSVA. Different from the GSEA method which depends on the ranked DEGs list and is limited to compare the different enriched pathways between different conditions, GSVA can display the pathways enriched patterns for each sample and can systematically identify the comparable pathways in different conditions. This analysis suggested that in addition to some similar functions, HuR in human and mouse may carry opposite regulatory function for certain essential pathways such as lipid metabolic process, cell cycle, and RNA processing (Figure 2I).

When the activity of a specific transcriptional or post-transcriptional factor was modulated under comparable in vitro or in vivo experiment settings for human and mouse liver cells, we persistently observed that a significant portion of regulated genes exhibit opposite responses, further supporting that they are very likely caused by intrinsic differences in gene regulation in human and mouse liver cells rather than by environmental factors or unwanted experimental variations.

### 3.3. Distinct Response in Gene Expression between Human and Mouse in the Chimeric Livers

In controlled interventional studies using a single specific treatment or genetic manipulation, we consistently observed a high percentage of differentially regulated genes between human and mouse. However, human and mouse liver cells in those studies are still in separated systems where experimental variations are inevitable. As the humanized mice described above have a chimeric liver, in which the mouse and human hepatocytes reside together in the same animal and share a common circulation, we reason that it would be an ideal in vivo system to directly compare gene regulation in human and mouse hepatocytes in response to identical physiological changes such as feeding cycle.

**Figure 3:**
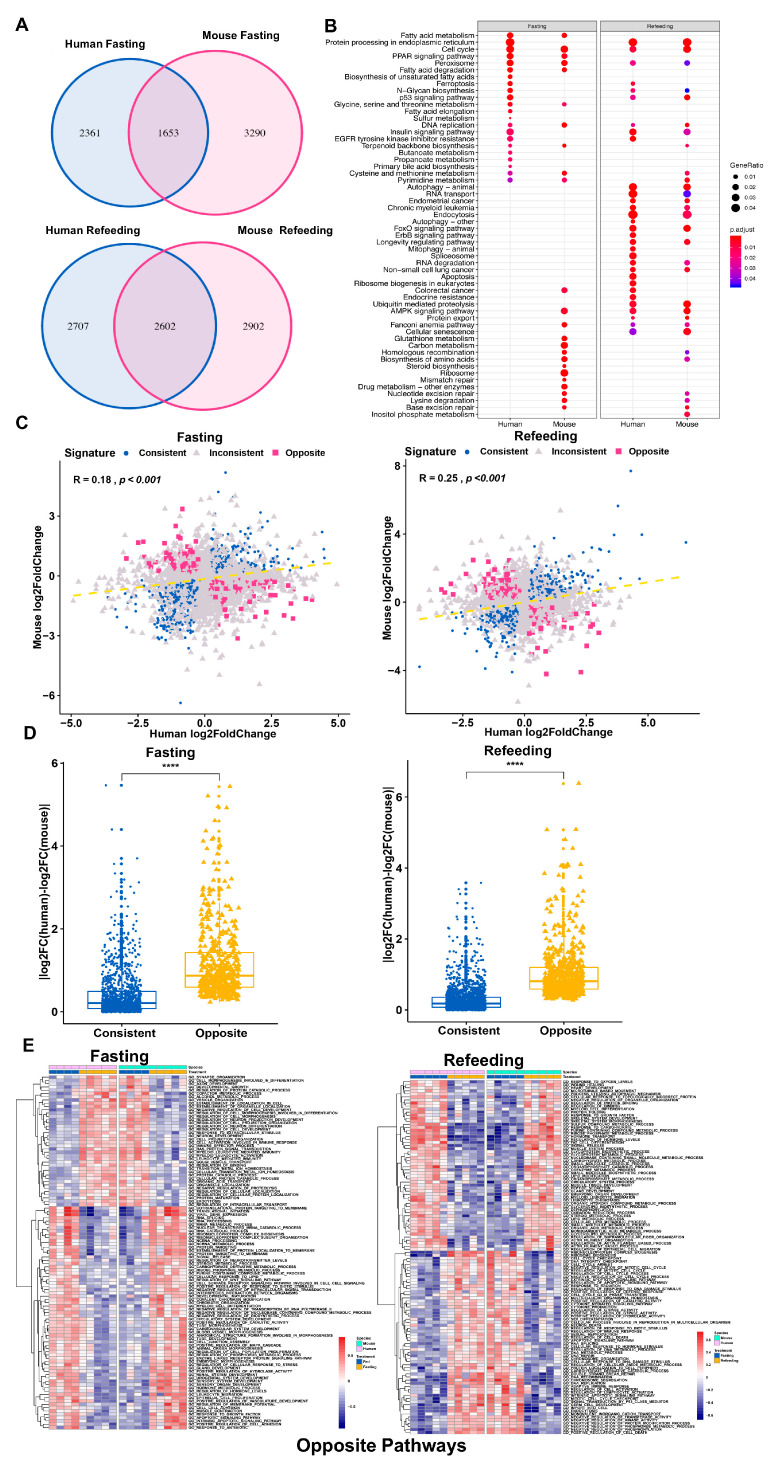
Distinct response in gene expression between human and mouse in the chimeric tissue (**A**) Venn diagram of the overlapped DEGs (*p* < 0.05) between human and mouse hepatocytes in chimeric humanized mouse liver under fasting and refeeding conditions, respectively. (**B**) Top enriched pathways for human and mouse DEGs in fasting and refeeding, respectively. (**C**) Correlation analysis of the log2 (fold change) for the human and mouse DEGs in the chimeric humanized mouse liver under fasting and refeeding conditions, respectively. (**D**) Divergence box plot for human and mouse DEGs in fasting and refeeding, respectively. Data represent mean ± SEM, **** *p* < 0.0001, two-tailed unpaired Student’s *t*-test. (**E**) GSVA enrichment score heatmap of human and mouse opposite DEGs in the chimeric humanized mouse liver under fasting and refeeding conditions, respectively.

We then performed RNA-seq on livers of humanized mice which were fed ad libitum, subject to a 24-h fasting or a 24-h fasting followed by a 5-h refeeding and analyzed the human and mouse transcriptomes separately. Interestingly, thousands of genes displayed inconsistent regulation between human and mouse even in the same in vivo context (Figure 3A). Furthermore, there were clearly distinctions between DEG enriched pathways in human and mouse during the fasting or refeeding state (Figure 3B).

We further examined the distribution of those commonly regulated genes between human and mouse, and found that there were 1181 consistent and 471 opposite genes in fasting compared to fed group, and 2012 consistent genes and 590 opposite genes in refeeding compared to the fasting group (Figure 3C). The consistent and opposite gene regulation pattern in human and mouse in both fasting and refeeding states is also manifested in the gene fold change divergency analysis (Figure 3D), as well as visualized in the heatmap (Figure S2A,B). Although, there were some consistently regulated genes and functional pathways in human and mouse (Figure S5A–D), the results of pathway enrichment analysis indicated opposite patterns in some metabolic pathways (Figure S5E,F).

We next performed GO term GSVA analysis on the oppositely regulated genes in human and mouse and found that they displayed very different enrichment patterns and some of the pathways showed opposite enrichment patterns in human and mouse (Figure 3E). For example, the human and mouse fasting opposite genes were oppositely enriched in the GO_RNA_SPLICING pathway (positive in human and negative in mouse), whereas the GO_LIPID_METABOLIC_PROCESS pathway was also opposite (positive in human and negative in mouse) during refeeding treatment.

Combining all conditions we tested, particularly in the chimeric humanized liver where the impact of most factors external to liver cells have been removed or minimized, we persistently observed a large number of oppositely regulated genes in human and mouse supporting they are an universal phenomenon and are most likely caused by cell-autonomous differences in gene regulation of human and mouse liver cells.

### 3.4. Nonconserved lncRNAs Might Contribute to the Divergent Gene Regulation between Human and Mouse

The divergent gene regulation under all conditions we tested support that there are many species-specific regulatory mechanisms and raises an intriguing question of how this regulation is encoded at the genomic level. In addition to transcriptional factors and post-transcriptional modulators, most of which are protein coding genes, mammalian genomes also harbor a large number of long noncoding RNAs (lncRNA) which have been shown to play a key role in regulating gene expression in recent years. Intriguingly, unlike protein coding genes, the conservation of lncRNAs between is very low [20,21] and only a very limited number of legitimate lncRNA homologs between human and mouse have been confirmed so far. Therefore, those species-specific lncRNAs that are coregulated with the protein coding genes could potentially contribute to the observed differences in gene expression between human and mouse.

**Figure 4:**
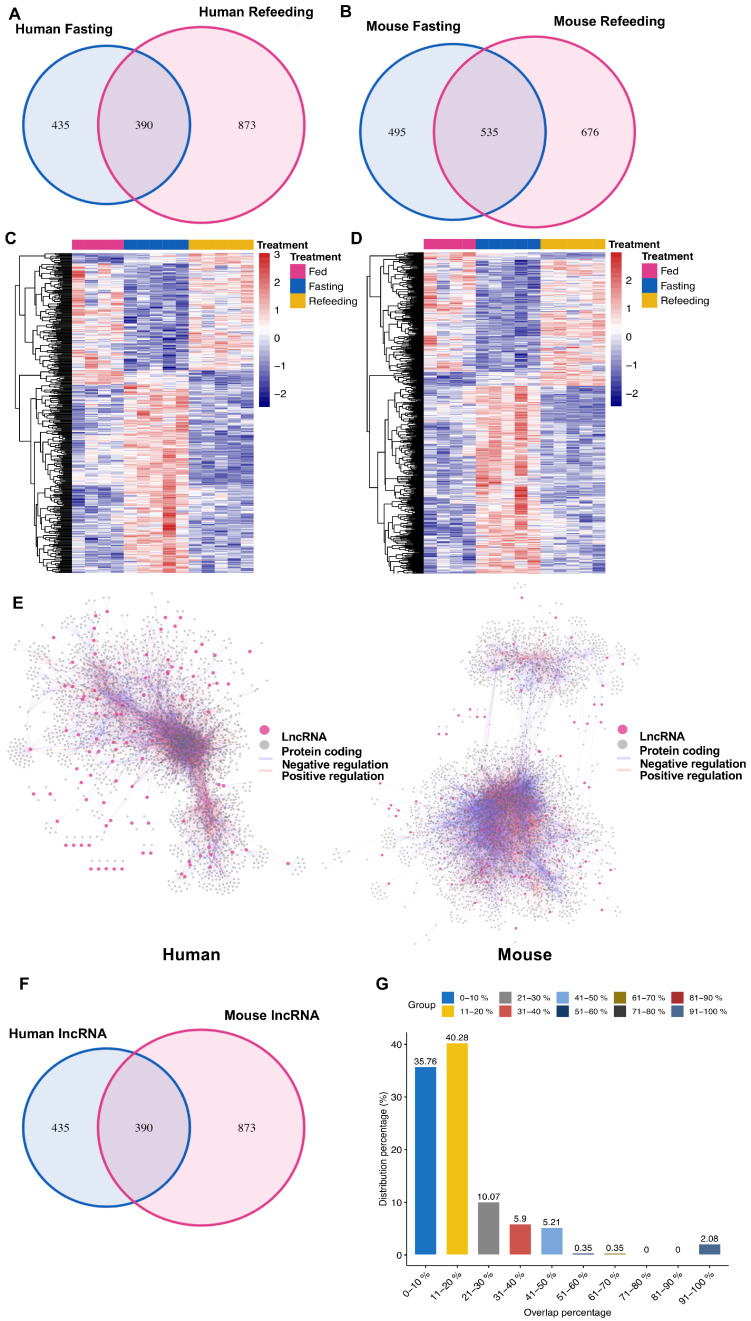
Nonconserved LncRNAs contribute to the divergent gene regulation between human and mouse. (**A**,**B**) Differentially expressed human and mouse lncRNA genes (*p* < 0.05) in the chimeric humanized mouse liver under fasting and refeeding conditions. (**C**,**D**) Heatmap displaying the expression levels of the human (**C**) and mouse (**D**) lncRNA genes changed in both of fasting and refeeding conditions in the humanized liver. (**E**) Correlation network of lncRNAs to protein coding genes in human and mouse. The connections passed |R^2^| > 0.9 and *p* < 0.001 are shown. (**F**) The overlap of human and mouse lncRNA DEGs showing some similarity in sequences with blast E value < 10^−5^ and length > 50 bp. (**G**) The correlated coding genes (|R^2^| > 0.9 and *p* < 0.001) for each changed human lncRNA were overlapped with the homologous mouse protein coding genes correlated with each changed mouse lncRNA, and the overlap percentages were calculated. The distribution of the maximum overlap percentage for the human lncRNAs are displayed.

To investigate this possibility, we further analyzed the regulated human and mouse lncRNA genes during fasting and refeeding in humanized mice. There were 390 human lncRNAs and 535 mouse lncRNAs which were regulated by both of fasting and refeeding (Figure 4A,B). These human and mouse lncRNAs in humanized mice displayed striking changes in their expression level during fasting and refeeding (Figure 4C,D). To check the connection of these lncRNAs to protein coding genes, we calculated the Pearson’s correlation between lncRNAs and protein coding genes in human and mouse, respectively, using corresponding expression data and we set the cutoff as |R^2^| > 0.9 and *p* value < 0.001 to visualize the network between lncRNAs and protein coding genes. As shown in Figure 4E, lncRNAs form integrated networks with protein coding genes suggesting that they are deeply involved in the regulation of these protein coding genes in human and mouse.

To explore the potential conservation of those regulated lncRNAs between human and mouse, we first compared their transcript sequences by Blast analysis. Strikingly, only five of 390 human lncRNAs transcriptional sequences could be blasted to mouse with e value < 10^−5^ and length >50, which indicated that these human and mouse lncRNAs exhibited poor sequence conservation (Figure 4F). Given it is challenging to infer the function of lncRNAs, which lacks information-rich open reading frames, a widely used method to connect lncRNAs to a specific function is to analyze their coexpressed protein coding genes. Thus, in order to determine the similarity of the functions of these regulated human and mouse lncRNAs, we identified the maximum percentage of overlapped coding genes which coexpressed with mouse or human lncRNAs in these humanized mice and the results showed that the overlap percentage of more than 75% mouse lncRNAs were below 20%, which suggested that human and mouse lncRNAs displayed very different functions (Figure 4G).

These data demonstrate that nonconserved human and mouse lncRNAs might play an important role in the complex cellular regulation network, and their functions could contribute substantially to the divergence in gene regulation between the two species.

## 4. Discussion

Mouse is the most widely used preclinical animal model yet the translational rate from mouse studies to therapies of human disease is dismal. In this report, we performed in-depth comparisons of gene regulation in human, mouse, and humanized livers and our results support that many genes and biological pathways exhibit species-specific regulation which need to be taken into consideration in mouse to human translation.

Our work addresses an important conceptual question and supports that a substantial fraction of differentially regulated genes between human and mouse livers are very similar to the results of cell autonomous mechanisms. We arrived at this conclusion by performing a series of comparative transcriptomics analyses between human and mouse livers to gradually remove the impact of non-cell-autonomous effects—basically a conclusion by exclusion approach. We first reduced the impact of genetic and gender biases and at the same time performed a rigorous comparison between human and mouse fatty liver disease in which we observed a near even split of consistently and oppositely regulated genes. We particularly focused on oppositely regulated genes as they were less likely to be results of experimental noises. Under well matched experimental conditions, we persistently identified that a sizeable fraction of all regulated genes (over 25%) go into opposite directions in human and mouse when a single specific transcriptional or post-transcriptional event was modulated. Most critically, in humanized mice in which most compounding factors that are external to liver cells are minimized, we also identified a high percentage of differentially regulated genes supporting that they are caused by cell-autonomous mechanisms. This notion that intrinsic differences in liver cells dictate many differential regulated genes in human and mouse has important implications, as it suggests that, although most protein coding genes are conserved in human and mouse, many human genes and biological pathways are subject to human-specific regulation and can only be faithfully studied in a human or humanized system. For example, it is well known that rodents are prone to the induction of peroxisome proliferation and the carcinogenic effects of fibrate drugs, the PPARa agonists, whereas humans are resistant to them [22,23]. The later developed PPARa-humanized mice significantly improved the understanding of the species difference mediated by PPARa [24].

Admittedly, our work has its limitations. First, although it is challenging to estimate the extent of comparability between human ethnicities and mouse strains, the human study we used, which only includes white individuals, might introduce ethnic bias and ideally a human study with a multiethnic human cohort should be used. Second, due to very limited availability and very high cost, humanized mice used in the study were generated with human primary hepatocytes from a small number of donors. Third, some genes in human hepatocytes might not fully maintain their expression levels in an immune-deficient system in humanized mice, although we did observe overall a robust correlation between humanized liver and human liver in gene expression. Fourth, only male and a small number of mice were used for most animal studies which might not be able to capture known sex differences in liver tissue [25,26,27]. Nevertheless, the humanized liver in which human and mouse hepatocytes share an identical in vivo environment represents the most closely matched in vivo condition. Furthermore, our work also set the stage for even more stringent comparisons in future with the development of an improved version of the humanized mouse model [28].

Our work also established a useful resource for studying the molecular underpinnings of human metabolic homeostasis and diseases. Due to the intrusive nature of liver biopsy, many aspects of our current understanding of human liver biology are based on extrapolation from mouse studies. Our work supports that the extent of differential gene expression could be far greater than what we have recognized and the utility of mouse-based studies in understanding human liver biology needs to be carefully scrutinized. In this regard, the extensive gene lists generated in our study could serve as critical reference sources for both basic and physician scientists. For example, our comparison of gene regulation by fatty liver disease without the impact of genetic and sex biases could serve as a more robust resource to evaluate the human relevance of pathophysiological mechanisms identified in mice. Furthermore, it has been increasingly recognized that metabolic responses are subject to extensive regulations at post-transcription level, but it is very challenging to carry out such studies in humans. Our gene profile in conventional and humanized mice with knockdown of HuR, an important post-transcriptional regulator that have been implicated in several aspects of energy metabolism [29,30,31], could provide many useful leads for post-transcriptional regulation in humans. It has been reported that around 25% of human and mouse transcripts exhibit different ARE (AU-rich element) patterns, which may be responsible for some of their divergent responses to HuR in these two species [32]. However, other mechanisms could also be involved. For example, we have recently identified a nonconserved human lncRNA regulating gene expression through HuR [11]. In this work, quite a number of nonconserved lncRNAs were also indeed identified, which might contribute to the divergent gene regulation between human and mouse. Finally, fasting and feeding are the extreme end of the caloric cycle and have been employed as a major tool to discover metabolism-related genes in mice but such a study cannot be done in healthy individuals and our work could fill in this void.

In summary, our work supports that many genes and pathways in human livers are subject to cell-autonomous species-specific regulation and our analyses also provide a timely resource for understanding the molecular basis of the similarity and divergency between human and mouse livers.

## Supplementary Materials

The following are available online at https://www.mdpi.com/2073-4409/9/12/2566/s1, Figure S1, Distinct gene regulation in model disease between human and mouse; Figure S2, Distinct response in gene expression between human and mouse under comparable conditions; Figure S3, Distinct response in gene expression between human and mouse under comparable; Figure S4, Distinct response in gene expression between human and mouse in the chimeric tissue; Figure S5, Distinct response in gene expression between human and mouse in the chimeric tissue; Table S1, List of the enriched pathways in the human NAFLD MEGENA modules; Table S2, List of the enriched pathways in the mouse HFD MEGENA modules; Table S3; List of the top 200 enriched pathways from GSEA GO term analysis in human and mouse; Table S4, List of sample information; Table S5, List of homology DEGs analysis results in human and mouse.
