# Supplementary material for: Comparative Transcriptomics Analyses in Livers of Mice, Humans, and Humanized Mice Define Human-Specific Gene Networks
Source: Cells. 2020 Nov 30;9(12):2566. doi: 10.3390/cells9122566 (PMC7761003; doi:10.3390/cells9122566)
Supplement: Supplementary file 1 [file cells-09-02566-s001.zip › supplement/Supplementary.pdf]

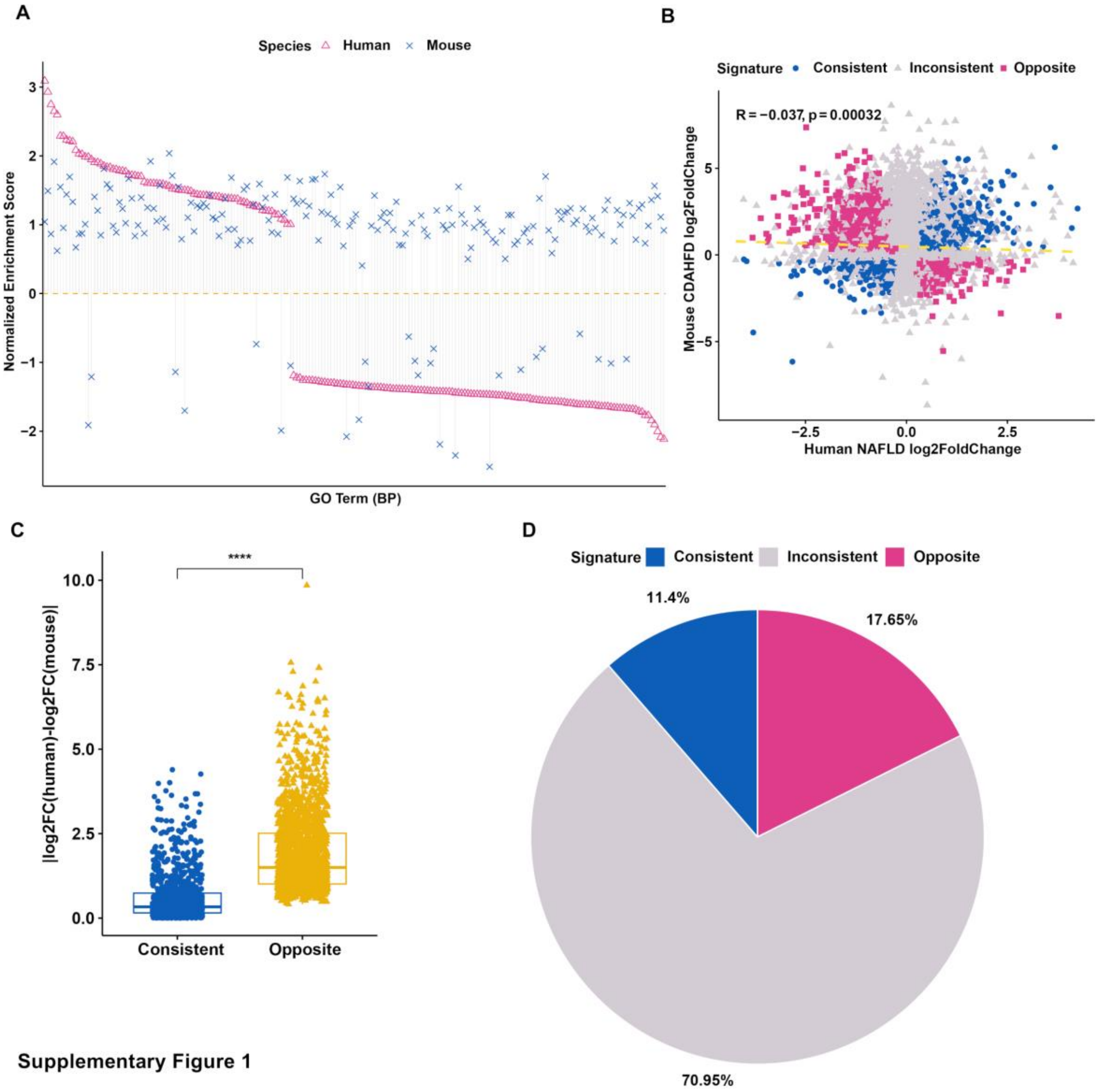

Supplementary Figure 1

Supplementary Figure 1. Distinct gene regulation in model disease between human and mouse (A) GSEA GO term analysis for more significant DEGs (padjust value<0.05, |logFC|>0.4) in human NAFLD and mouse CDAHFD treatment, showing the shared terms. (B) Correlation analysis of the log2(fold change) for the DEGs (padjust value <0.05) in human and mouse fatty liver disease. Consistent genes mean log2(fold change) human * log2(fold change) mouse>0; Opposite genes mean log2(fold change) human * log2(fold change) mouse<0; Inconsistent genes are only differentially expressed either in human or mouse fatty liver disease. (C) The divergence (|log2(human fold change)-log2(mouse fold change)|) of log2(fold change) values for consistent and opposite genes between human and mouse. Data represent mean ± SEM, **** $p < 0.0001$, two-tailed unpaired Student's t-test. (D) The percentages of consistent, opposite and inconsistent genes.

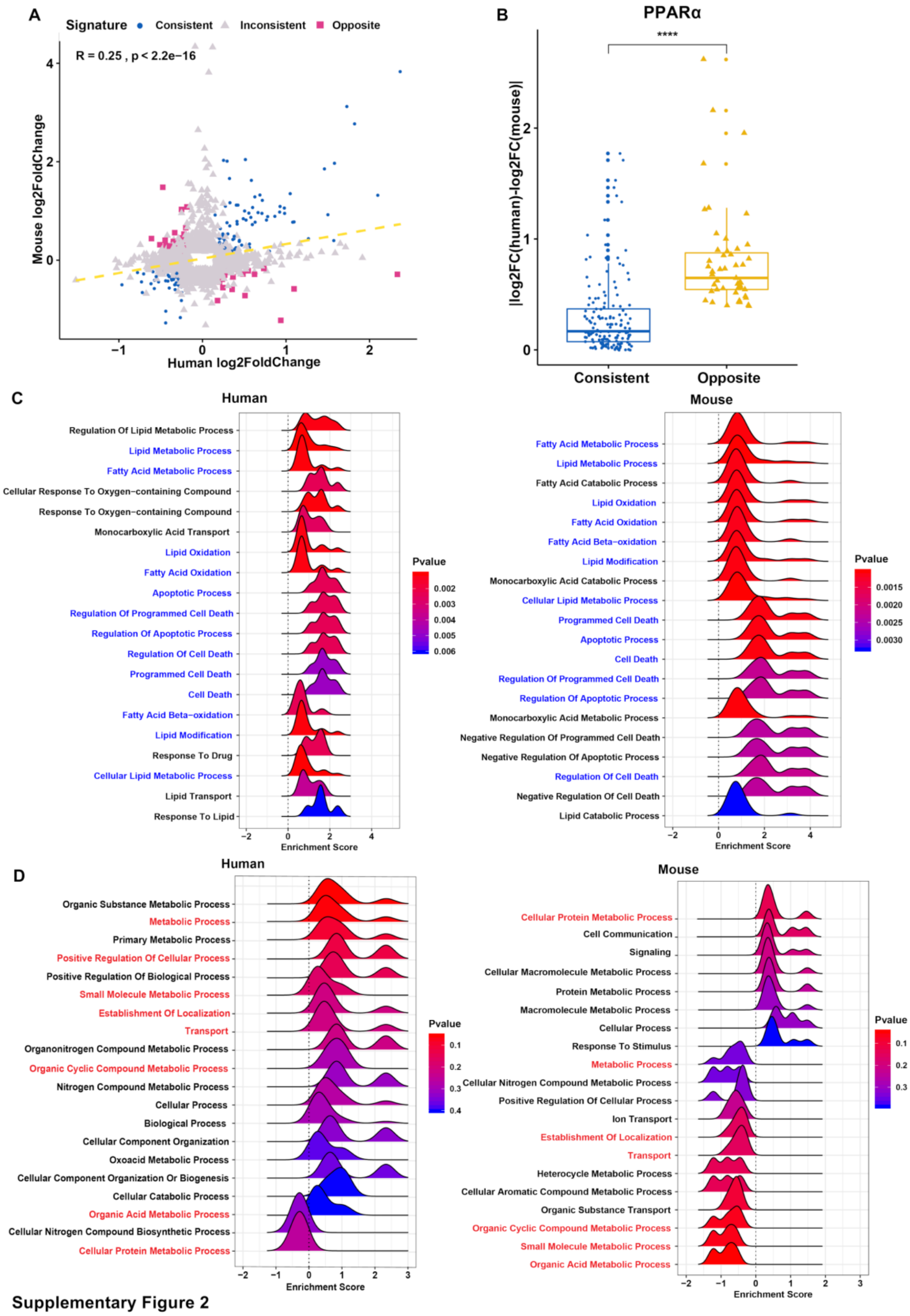

Supplementary Figure 2

Supplementary Figure 2. Distinct response in gene expression between human and mouse under comparable conditions (A) Correlation analysis of the log2(fold change) for DEGs induced by PPAR*α* agonist treatment in human and mouse primary hepatocytes. (B) Divergence box plots for human and mouse consistent and opposite DEGs associated with PPAR*α* agonist treatment. (C, D) GSEA running enrichment score ridge plots of the enriched GO term pathways for consistent or opposite DEGs with PPAR*α* agonist treatment in human and mouse primary hepatocytes, respectively. The pathways with consistent enriched direction were labelled as blue and the ones with opposite direction were labelled as red.

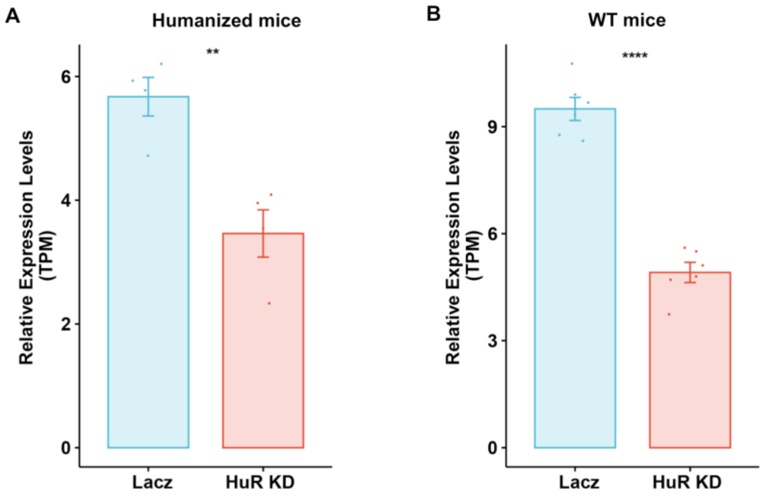

Supplementary Figure 3. Distinct response in gene expression between human and mouse under comparable conditions (A, B) HuR expression levels of Lacz and HuR KD group in humanized and WT mice livers. Data represent mean ± SEM, **p<0.01; **** p < 0.0001, two-tailed unpaired Student's t-test.

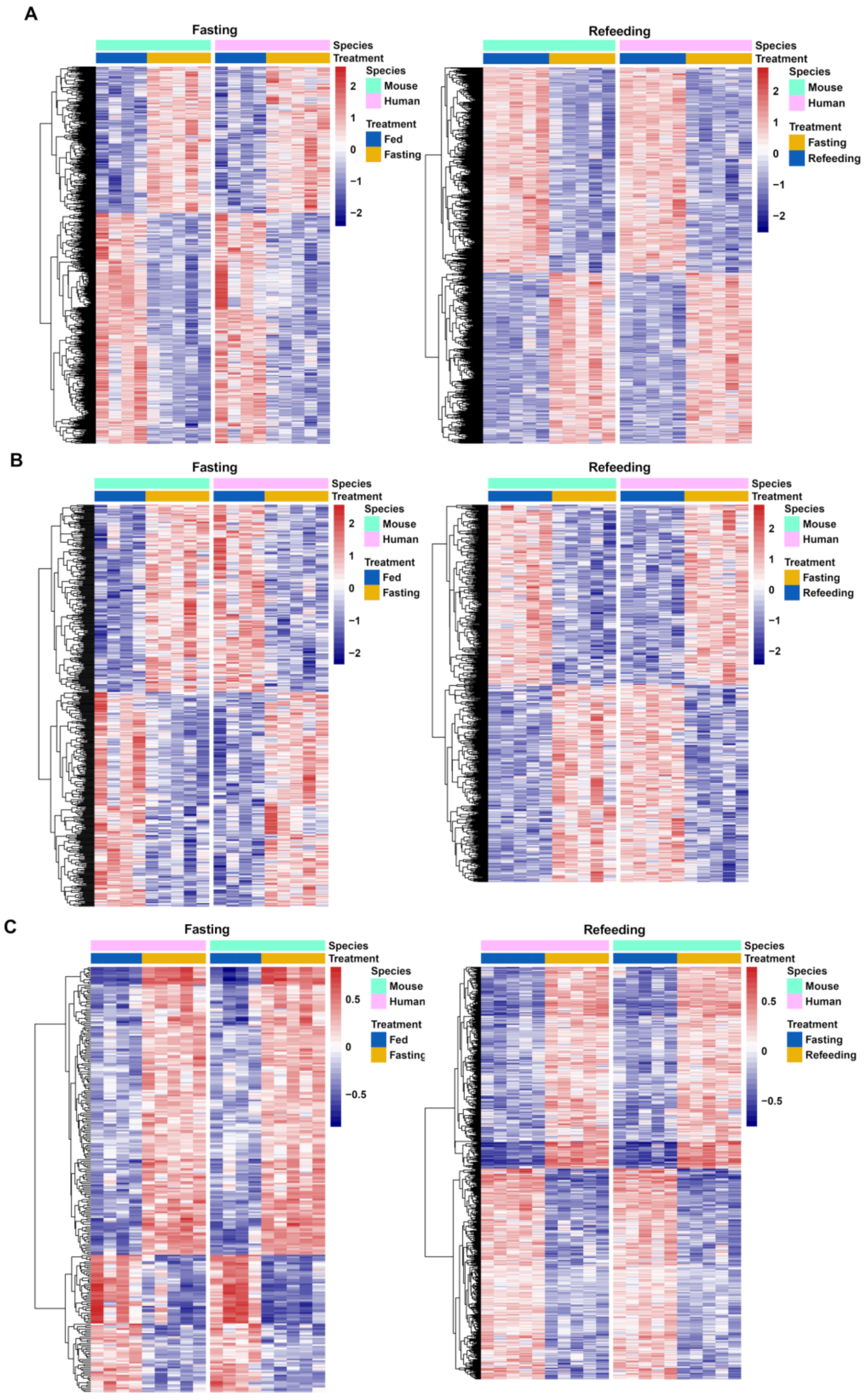

Supplementary Figure 4

Supplementary Figure 4. Distinct response in gene expression between human and mouse in the chimeric tissue (A, B) Expression level heatmaps of human and mouse consistent and opposite gene in fasting and refeeding conditions. (C) GSVA enrichment score heatmap of human and mouse opposite genes in humanized mice under fasting and refeeding conditions.

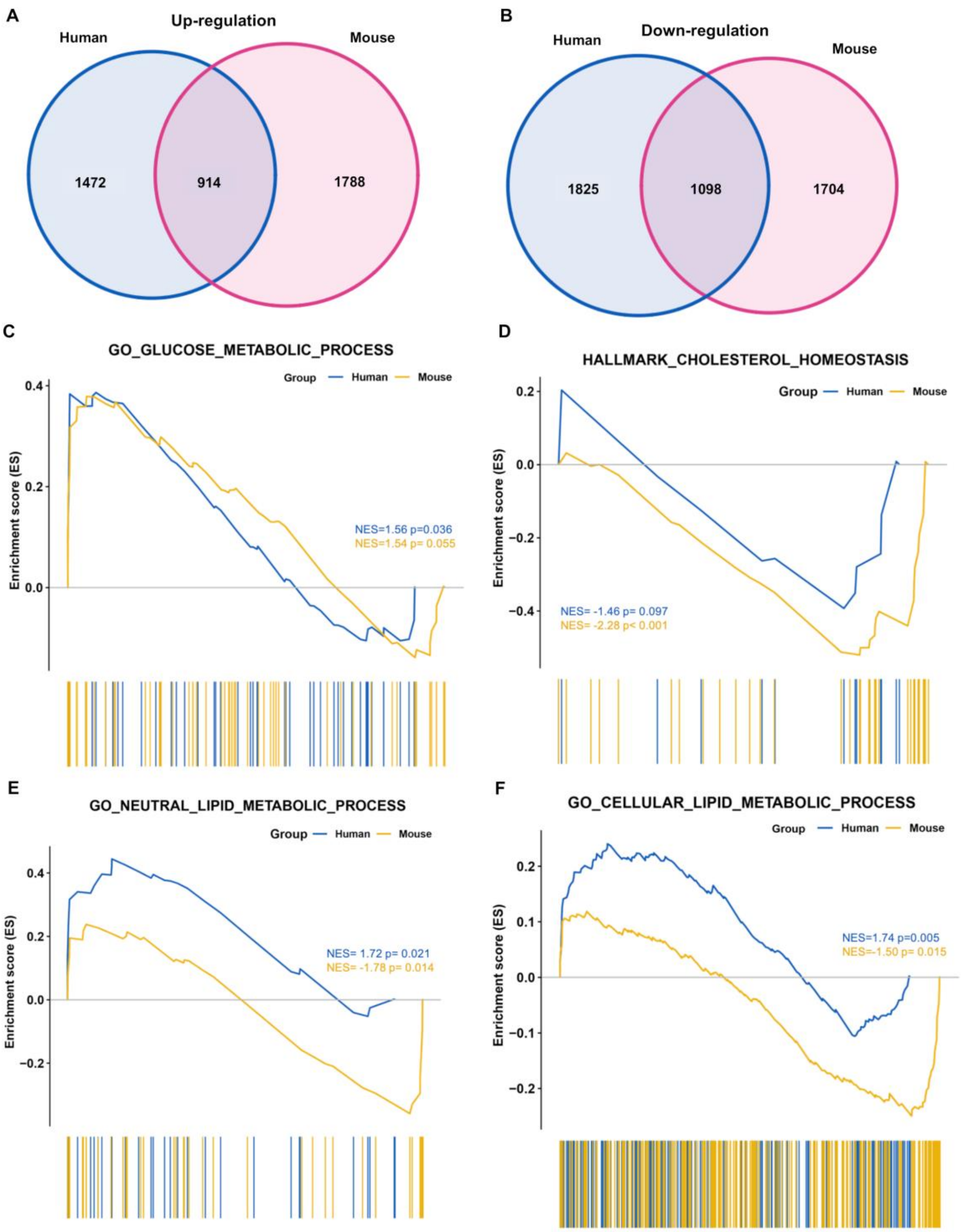

Supplementary Figure 5. Distinct response in gene expression between human and mouse in the chimeric tissue (A, B) Venn Diagram of commonly up-regulated and down-regulated genes by refeeding in the chimeric tissue. (C-E) GSEA analysis for metabolic pathways using human and mouse refeeding DEGs (padjust value<0.05, |logFC|>0.4) in the chimeric liver.

Supplemental Table 1. List of the enriched pathways in the human NAFLD MEGENA modules.

Supplemental Table 2. List of the enriched pathways in the mouse HFD MEGENA modules.

Supplemental Table 3. List of the top 200 enriched pathways from GSEA GO term analysis in human and mouse.

Supplemental Table 4. List of sample information.

Supplemental Table 5. List of homology DEGs analysis results in human and mouse.
